# Alveolar Soft Part Sarcoma of Urinary Bladder Occurring as a Second Primary Malignancy: A Case Report and Literature Review

**DOI:** 10.1155/2016/4746061

**Published:** 2016-07-28

**Authors:** Manint Usawachintachit, Piyada Sitthideatphaiboon, Voranuch Thanakit, Sulada Pukiat, Kamol Panumatrassamee, Julin Opanuraks, Apirak Santi-Ngamkun

**Affiliations:** ^1^Division of Urology, Faculty of Medicine, Chulalongkorn University and King Chulalongkorn Memorial Hospital, The Thai Red Cross Society, Rama IV Road, Patumwan, Bangkok 10330, Thailand; ^2^Division of Medical Oncology, Department of Internal Medicine, Faculty of Medicine, Chulalongkorn University and King Chulalongkorn Memorial Hospital, The Thai Red Cross Society, Rama IV Road, Patumwan, Bangkok 10330, Thailand; ^3^Department of Pathology, Faculty of Medicine, Chulalongkorn University, Patumwan, Bangkok 10330, Thailand; ^4^Division of Hematology, Department of Medicine, Faculty of Medicine, Ramathibodi Hospital, Mahidol University, 270 Rama VI Road, Ratchatewi, Bangkok 10400, Thailand

## Abstract

We report a case of bladder alveolar soft part sarcoma in an 18-year-old Thai male patient who had been treated with testicular radiation and systemic chemotherapy for acute lymphoblastic leukemia with testicular relapse. He presented with recurrent dysuria and gross hematuria. Cystoscopy revealed a 2-centimeter irregular sessile mass at the bladder base adjacent to left ureteral orifice. Transurethral resection of the tumor was performed. The histopathological diagnosis was alveolar soft part sarcoma. Chest and abdominal computed tomography showed no evidence of metastasis. He was treated with partial cystectomy and left ureteral reimplantation with negative surgical margin. No evidence of recurrence was found during a 28-month follow-up period with surveillance cystoscopy and computed tomography of the chest and abdomen.

## 1. Introduction

Alveolar soft part sarcoma (ASPS) is a rare soft tissue neoplasm, accounting for 0.5 to 1% of all soft tissue sarcomas. It typically arises in the deep soft tissue of buttock and thigh but rarely grows in the genitourinary system. Herein we report a case of alveolar soft part sarcoma arising in the urinary bladder, which to our knowledge is the first reported case that occurred as a second primary tumor following radiation therapy.

## 2. Case Presentation

An 18-year-old Thai male patient presented with recurrent painful hematuria over a year. He was initially treated as urinary tract infection but did not respond to several courses of antibiotics. Later, a renal and bladder ultrasound revealed 1.7 × 1.1 cm hyperechoic nodule at the left lateral aspect of his urinary bladder. Subsequent computed tomography demonstrated normal upper urinary tracts and no lymphadenopathy. He was diagnosed with acute lymphoblastic leukemia (ALL) at the age of 4 and treated with systemic chemotherapy. Bone marrow and extramedullary relapse in the testis occurred few years after treatment completion. Salvage chemotherapy and testicular radiation led to complete remission when he was 11 years old.

**Figure 1:**
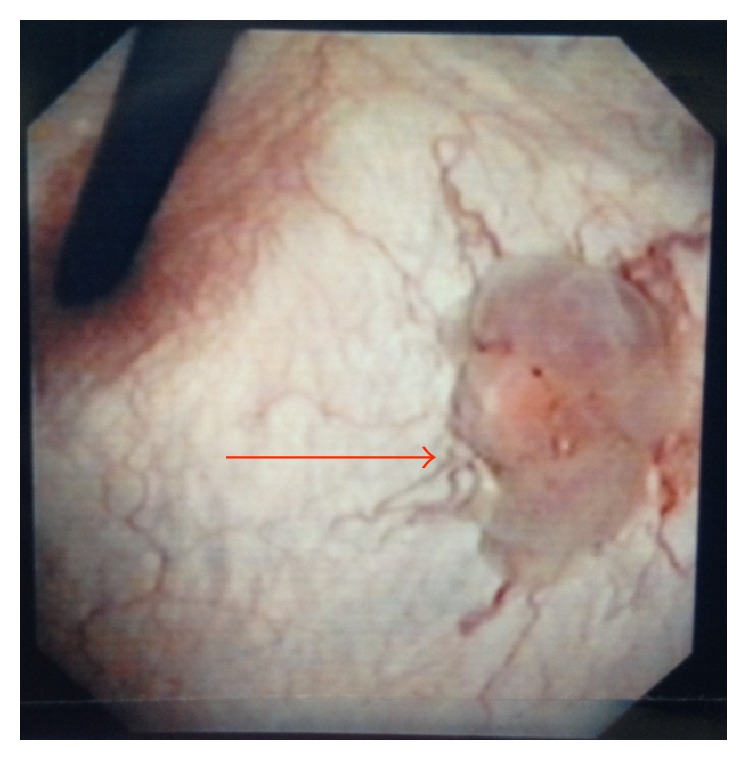
Initial flexible cystoscopic finding. A two-centimeter nonpapillary bladder mass was found at the bladder base with surrounding hypervascularization (arrow).

**Figure 2:**
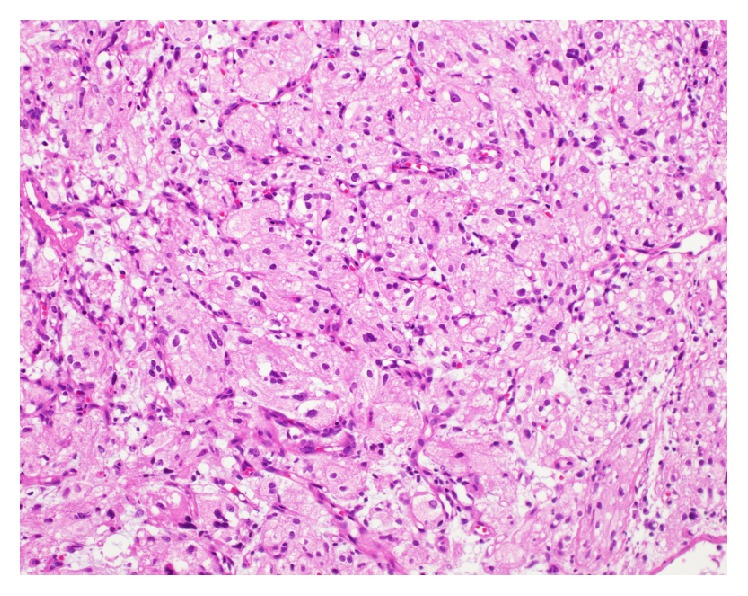
H&E staining on low-powered field. The infiltrative tumor composed of polygonal cells, which arranged in small nests and intervened with blood vessels. These tumor cells posed abundant finely granular eosinophilic to foamy cytoplasm and moderately irregular nuclei.

**Figure 3:**
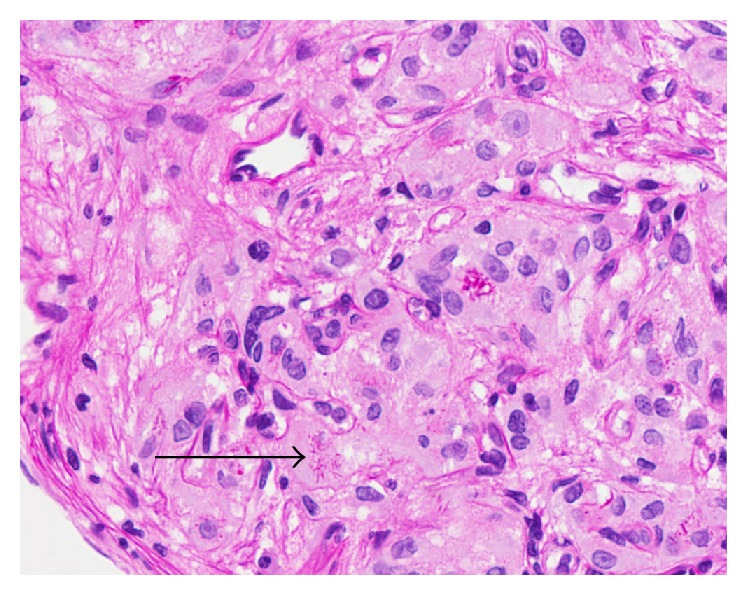
Periodic acid Schiff (PAS) staining on high-powered field. The tumor showed positive stain and it demonstrated characteristic diastase-resistant crystalline structures with rhomboid and rod-like structure in some tumor cells (arrow).

**Figure 4:**
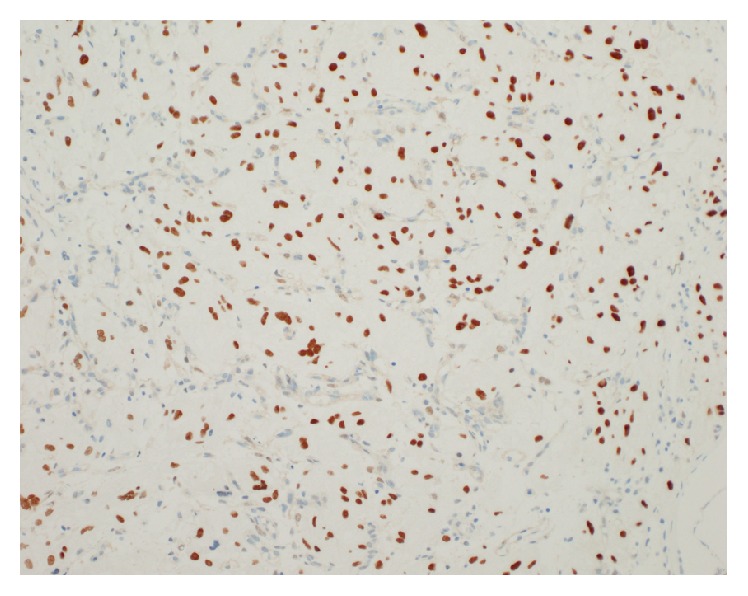
Transcription factor E3 (TFE3) staining on medium-powered field. The tumor showed uniform, strong nuclear positivity against the C-terminus of TFE3.

Upon presentation to our department, cystoscopy demonstrated a friable two-centimeter broad-based nodule at the bladder base adjacent to the left ureteral orifice with surrounding hypervascularization (Figure 1). A transurethral resection was performed. Histopathological examination demonstrated the infiltrative lesion in the lamina propria without dysplasia of the overlying urothelium. The tumor was composed of polygonal cells arranged in small nests intervening with blood vessels. These cells posed abundant fine granular eosinophilic to foamy cytoplasm and irregular nuclei (Figure 2). Immunohistochemical (IHC) staining revealed periodic acid Schiff- (PAS-) positive and diastase-resistant crystalline structures with rhomboid and rod-like structure of some tumor cells (Figure 3), diffuse and strongly positive for CD 10 and TFE3 (Figure 4) but negative for AE1/AE 3, CAM5.2, cytokeratin 34*β*E12, vimentin, RCC, p504s, desmin, S-100, HMB45, melan-A, chromogranin-A, CD 34, and CD 68. A diagnosis of alveolar soft part sarcoma was established based on the compatible histomorphological finding, presence of crystal, and the positivity of TFE3 on IHC staining and by exclusion of other bladder tumors.

The patient underwent partial cystectomy with left ureteral reimplantation. Open midline transperitoneal approach was selected. Part of left-sided bladder wall and left distal ureter was removed en bloc. Transvesical technique was applied for left ureteral reimplantation. Pelvic lymph node dissection was performed with a standard template. Postoperative course was uneventful with a hospital stay of 5 days. The histopathological finding demonstrated benign urothelium lining with underlying edematous stroma and focal foreign body granulomas. No residual tumor was documented. Six reactive lymph nodes were found without evidence of tumor.

After the discussion in our multidisciplinary tumor board, we opted for clinical observation and regular surveillance without adjuvant therapy for this patient. Subsequent surveillance consisted of periodic cystoscopy along with chest and abdominal computed tomography. Until now, no evidence of local recurrence or distant metastasis is detected during a 28-month follow-up duration.

## 3. Discussion

The entity of alveolar soft part sarcoma (ASPS) was first described by Christopherson et al. in 1952 [1], although several cases might have been previously reported under various entities of pathological diagnosis such as “malignant myoblastoma” and “malignant granular cell myoblastoma” [2]. Most cases occur in the extremities and buttock and chest wall. ASPS predominantly affects adolescents and young adults and tends to have late metastasis [3]. In the genitourinary system, there have been some case reports of ASPS involving uterine corpus, kidney, and prostate [4, 5].

ASPS of the urinary bladder is exceedingly rare with 3 reports in the literature. The first published case was a 31-year-old male presenting with hematuria and large pelvic mass [6]. Tumor resection and partial removal of bladder were performed and histopathological finding showed a tumor with characteristic granular cytoplasm, and a diagnosis of “malignant granular cell myoblastoma” was concluded. The tumor locally recurred rapidly and the patient died 17 months after the operation.

The second case was a 37-year-old male presenting with a slow growing suprapubic mass and bilateral ureteral obstruction [7]. Subsequent biopsy confirmed a diagnosis of ASPS by electron microscopy, but the authors mentioned neither the treatment nor the clinical course of this patient. The last published case was a 25-year-old female patient presenting with dysuria and hematuria [8]. Cystoscopy revealed a tumor located at the bladder trigone and a diagnosis of ASPS was given by histopathologic finding from transurethral tumor resection. Subsequently, this patient noticed a rapid growing painful mass protruding from her urethral meatus. This tumor was resected en bloc and no additional therapy was administered. A follow-up of 45 months after the initial presentation showed no evidence of recurrence.

Given the paucity of ASPS cases, it remains unclear whether surgery alone or surgery with addition therapy is superior to others. As in other soft tissue sarcomas, radical surgery is the main therapy for localized disease with an ultimate goal of achieving R0 resection. Most series in other soft tissue ASPS have suggested that this tumor is relatively resistant to conventional cytotoxic chemotherapy [9]. However, there is some evidence supporting the use of antiangiogenic agent in advanced ASPS due to the highly vascularized nature of this tumor [10].

Our case represents the first case of bladder ASPS arising as a second primary malignancy. This patient was treated successfully with organ-preserving surgery and close observation. Although the risk factor predisposing to ASPS is unknown, prior exposure to radiation therapy and cytotoxic chemotherapy in this patient may play some roles in carcinogenesis. His bladder ASPS has met all criteria for diagnosis of postradiation sarcoma defined by Cahan et al. as follows: (1) lesions of different histologic features to that of the original lesion, (2) being located within the field of radiation, and (3) arising after a latency period of more than four years [11]. This patient had received testicular radiation for salvage treatment of leukemia, the radiation field was close to bladder area, and bladder ASPS was found 7 years after the completion of radiation which fit all of these proposed criteria. Another issue is to examine the genetics of postradiation sarcomas, which may be different from sporadic cases. But, until now, very little is known about the genetic changes involved in this specific type of sarcomas. Exposure to multiagent chemotherapy, particularly alkylating agents, may potentiate the effect of previous radiation therapy in childhood and thus serve as another predisposing factor for the development of a postradiation sarcoma [12]. Presumably, there might be a genetic predisposition that links acute leukemia and soft tissue sarcoma, which has not yet been discovered.

In conclusion, the optimal treatment for bladder ASPS has to be further defined. Radical resection remains the therapy of choice for localized disease, but the organ-preserving surgery with stringent follow-up in selected patient is a reasonable option, which may improve the quality of life without compromising clinical outcomes.
